# The impact of coronavirus disease 2019 (COVID-19) on provider use of electronic hand hygiene monitoring technology

**DOI:** 10.1017/ice.2020.1336

**Published:** 2020-11-20

**Authors:** Olivia C. R. Hess, Jo Dee Armstrong-Novak, Michelle Doll, Kaila Cooper, Pamela Bailey, Emily Godbout, Michael P. Stevens, Gonzalo Bearman

**Affiliations:** Healthcare Infection Prevention Program, Virginia Commonwealth University Health System, Richmond, Virginia

## Abstract

The use of an electronic hand hygiene monitoring system (EHHMS) decreased due to the coronavirus disease 2019 (COVID-19) pandemic. We analyzed dispenser use, hand hygiene (HH) badge use, and HH compliance to determine the effect of COVID-19 on EHHMS use and HH compliance. HH product shortages and other pandemic-induced challenges influenced EHHMS use.

Hand hygiene (HH), the foundation of infection prevention,^1^ is important both in the context of the coronavirus disease 2019 (COVID-19) pandemic and as it relates to concerns about viral contamination of the inanimate environment.^2,3^ However, the pandemic has resulted in shortages of alcohol-based hand sanitizers as well as changes in personal protective equipment (PPE) donning and doffing procedures.^4^ In 2019, our institution introduced an electronic HH monitoring system to promote behavioral changes for improved HH compliance.^5,6^ However, the use of HH monitoring badges within our institution decreased during the COVID-19 pandemic.

## Methods

An electronic HH monitoring system (EHHMS) was installed in the inpatient areas of the hospital in May 2019. The technology uses personally identifiable badges worn by healthcare workers. Each badge records individual HH events through a wireless connection with sensors in soap and alcohol-based product dispensers. Beacons attached to patient beds monitor HH compliance before and after patient care. Dispenser use by badged and unbadged staff, patients, and visitors was recorded.

The distribution of badges to staff is underway in a phased implementation; units and provider groups have been assigned to 1 of 5 waves. The first implementation wave took place in May 2019 (9 units), the second in October 2019 (7 units), and the third in January 2020 (6 units). The pandemic interrupted plans to implement waves 4 and 5 in April and July, respectively. COVID-19 patients were primarily housed on 3 units within the hospital: a progressive care unit, an acute-care unit, and the medical respiratory intensive care unit (MRICU). The progressive care unit was issued badges in wave 1. The acute-care unit and the MRICU received badges in wave 2.

In addition to the EHHMS, HH observers make rounds on units to monitor HH compliance. Trained observers assess HH compliance in a standardized manner, as previously described.^7^ Their work continued uninterrupted by the COVID-19 pandemic.

The pandemic caused increased demand for sanitizer resulting in HH product shortages. We began allocating our HH product in February 2020 and subsequently rationed the wall-dispenser sanitizer at the beginning of March 2020. When a unit ran out of sanitizer in a wall dispenser, they were supplied with pump-bottle sanitizers or flip-top bottles. The wall dispenser was unavailable for a short period starting April 18, 2020. During this period, each unit was supplied with a maximum of 2 pump bottles of sanitizer per day. On April 28, 2020, we began reusing and refilling wall-sanitizer bottles with an alternate sanitizer product.

The first COVID-19 patient under investigation (PUI) was seen in our hospital on March 2, 2020. For the purposes of this study, we consider November 2019 to February 2020 our pre–COVID-19 period and March 2020 to June 2020 our post–COVID-19 period. COVID-19 volume in the hospital is expressed as COVID-19 patient days of all patient days for the following months: March (117 of 18,858), April (861 of 14,297), May (1,158 of 17,873), and June (959 of 18,591). The COVID-19–positive patient rooms required distinct procedures for entering and exiting with PPE. However, badges (including ID and HH badges) were worn under the PPE throughout the patient care episode, such that no specific treatment was required for badges after doffing PPE unless a breach occurred. To better understand the trends in use of the EHHMS and to identify barriers to use, we analyzed monthly HH data from both the observers and the EHHMS. Variables of interest included compliance through the EHHMS and direct observation, HH product use through the EHHMS, badge use by providers, and location (COVID-19 unit vs non–COVID-19 unit). Pre– and post–COVID-19 data were compared, as was practice on COVID-19 units versus non–COVID-19 units. Compliance data were analyzed using χ^2^ tests using Excel (2016, Microsoft, Redmond, WA).

## Results

### HH product use

Dispenser use reached a peak in March with a 17% increase from November from 2,058,977 to 2,403,199 dispensing events (Fig. 1a). Dispenser use by badged users also increased 37% from November to March, from 650,963 to 889,300 dispensing events. After March, overall dispensing events decreased by 20% (from 2,403,199 in March to 1,918,901 in June). Badged dispensing events decreased by 63% (from 889,300 in March to 332,911 in June).

**Fig. 1a.**
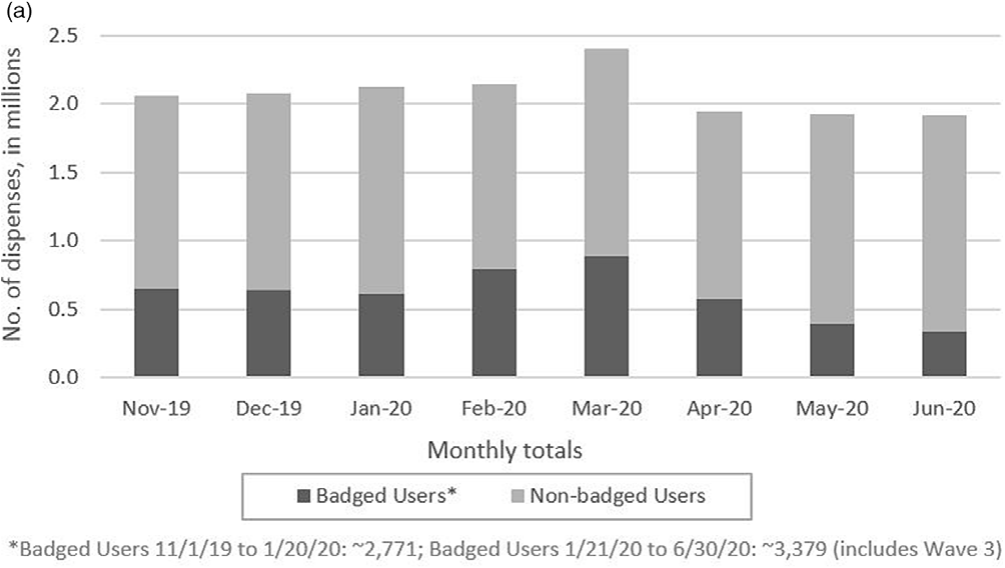
Hospital-wide Soap and Alcohol-based Hand Rub Dispenser Usage

### Badge use

Before the COVID-19 pandemic, an average of 453 wave 1 and 2 employees used their badges per day. After the COVID-19 pandemic, the average number declined by 44% (427 in February to 240 in June). On COVID-19 units, the average daily number of nursing staff using their badges went from 64 in February to 21 in June, a 67% decline in use (Fig. 1b). Wave 3 units are not included in these analyses because technology practices were not firmly established on those units prior to the pandemic.

**Fig. 1b.**
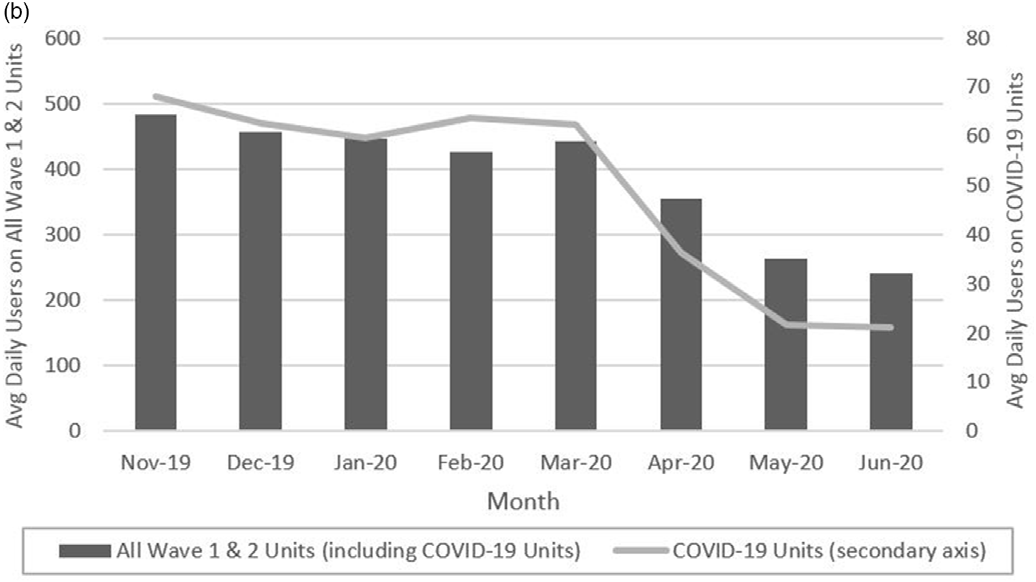
Average Daily Electronic Hand Hygiene Monitoring System Badge Users

### HH compliance

Direct observation data indicated an increase in compliance of 10% on COVID-19 units (*P* < .0001) and 6% hospital-wide when comparing pre– versus post–COVID-19 data (*P* < .0001). The EHHMS data showed an increase in compliance of 1% on both COVID-19 units (*P* = .0002) and hospital-wide (*P* < .0001) when comparing pre– versus post–COVID-19 data (Table 1). HH observation opportunities decreased for both observation methods (Table 1).

**Table 1.** Hand Hygiene Compliance Data from Direct Observation and Electronic Hand Hygiene Monitoring System Data

		Direct Observation Data			Electronic Hand Hygiene Monitoring System Data		
		No. of Hand Hygiene events	No. of Hand Hygiene opportunities	Hand Hygiene compliance, % of opportunities	No. of Hand Hygiene events	No. of Hand Hygiene opportunities	Hand Hygiene compliance, % of opportunities
**COVID-19 Units**							
	Pre-COVID-19 (Nov 2019 - Feb 2020)	2,255	2,834	80%	188,086	210,530	89%
	Post-COVID-19 (Mar 2020 - Jun 2020)	1,358	1,504	90%	50,176	55,826	90%
				p<0.0001			p=0.0002
**Hospital-wide**							
	Pre-COVID-19 (Nov 2019 - Feb 2020)	17,525	22,639	77%	1,034,024	1,229,292	84%
	Post-COVID-19 (Mar 2020 - Jun 2020)	13,899	16,710	83%	441,093	519,078	85%
				p<0.0001			p<0.0001

NOTE: Direct observation data includes physicians, housestaff, nursing staff, and patient & support services. EHHMS on COVID-19 units includes nursing staff. EHHMS hospital-wide includes physicians, housestaff, nursing staff, and patient & support services.

## Discussion

The COVID-19 pandemic presented new barriers to HH compliance and the use of an EHHMS. Lack of access to wall sanitizer may have affected healthcare workers ability to use foam on entry and exit using the technology. Use of badges decreased due to perceived inconvenience and incompatibility with donning and doffing procedures for COVID-19 patient rooms. Staff expressed concerns about potentially contaminating items taken into rooms and the time-consuming nature of cleaning the badge after leaving each COVID-19 room. Suboptimal messaging regarding badge best practices played a significant role in the decrease of badge use throughout the hospital. Badge users also reported a perception that the EHHMS was no longer necessary or accurate given the introduction of alternate products during allocation and shortages of wall sanitizer. Due to the constant changes in the environment during the pandemic, monthly compliance reports were not distributed.

Nevertheless, overall HH compliance by direct observation increased during the COVID-19 pandemic, particularly on COVID-19 patient units. An overall increase in HH despite declining usership of EHHMS badges is indicated by the dispensing events recorded by the EHHMS. The number of dispensing events increased in March, followed by relative stability in April and May despite a lack of visitors in the environment using the dispensers, similar to that reported by Moore et al.^8^ This finding suggests more events by bedside staff during this time period whether or not they were badged. The decrease in opportunities for HH measured by both observers and the EHHMS is likely the result of a lower overall census and limitations on room entries during the COVID-19 pandemic.

HH is recognized as important by healthcare providers during the COVID-19 pandemic, particularly on high-risk units. However, barriers to badge use may prevent participation in the EHHMS, which has the ability to provide an extra layer of safety in the form of end-user reminders. Confusion around management of the badge when entering and exiting rooms highlights a need to address other items such as ID badges and cell phones in PPE procedures such that providers understand best practices for protecting these items from contamination and disinfecting them routinely. Product shortages are disruptive when an infection prevention program is working to optimize infection prevention initiatives. Significant efforts will need to be directed toward re-establishing the EHHMS as an integral component of the organizational safety mission.
